# The new frame for Mucopolysaccharidoses

**DOI:** 10.1186/s13052-018-0549-y

**Published:** 2018-11-16

**Authors:** Rossella Parini, Andrea Biondi

**Affiliations:** 1 Pediatric Clinic, Fondazione MBBM, Ospedale San Gerardo, via Pergolesi 33, Monza, Italy; 2 San Raffaele Telethon Institute for Gene Therapy (SR-TIGET), IRCCS San Raffaele Scientific Institute, Milan, Italy

## Abstract

Mucopolysaccharidoses (MPS) are genetic, progressive, lysosomal storage disorders affecting virtually all organs and systems. The first MPS were clinically identified about 100 years ago. Nowadays, the enzyme defects and related genes are known for all 11 different enzyme defects. Treatments are available for many MPS but these have only partial efficacy, especially when started late. The problems to solve are: 1) the need for an earlier diagnosis (neonatal screening? improving the awareness of physicians?); 2) prompt access to therapies; 3) improving the efficacy of the available treatments; 4) finding new treatments; and 5) the availability of specialist experts in MPS who can meet the traditional needs of MPS patients. This introduction to the IJP Supplement on MPS is a brief comment on the different papers accepted for this volume, which are in turn the elaboration of the lectures given at a meeting on the future of mucopolysaccharidoses held in Milan on 8–9 May 2017.

## Introduction

**Fig. 1:**
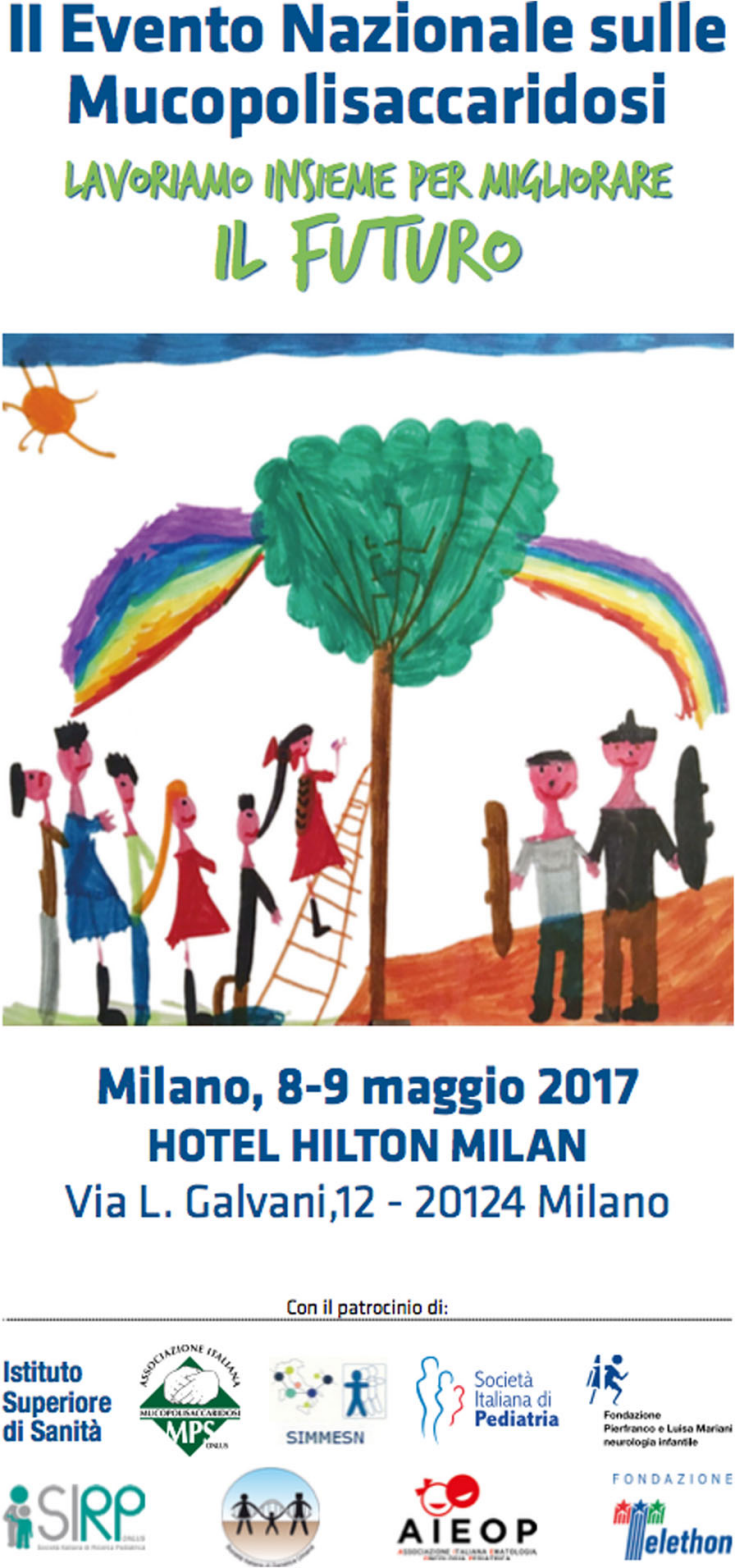
The leaflet from the meeting on mucopolysaccharidosis held in Milan 8–9 May 2017

This supplemental issue of the *Italian Journal of Pediatrics* presents relevant reviews on the clinical presentation, biochemical and molecular diagnosis, and palliative and etiologic treatments of mucopolysaccharidoses (MPS), and future directions for their diagnosis and treatment. We decided to collect all these reviews into one issue during a meeting on MPS which was organized in Milan on 8–9 May 2017 (Fig. 1) with the sponsorship of many institutions and the financial support of BioMarin, Sanofi Genzyme, Shire, Ultragenyx and Chiesi. Our aim is to provide, in a succinct but not trivial manner, useful information to clinicians already caring for individuals with MPS and those who do not follow MPS patients and who want to become acquainted in their diagnosis and treatment. In this brief preface, we will introduce the articles in the Supplement and make a few comments on the current challenges seen in MPS disorders.

## History of MPS

MPS are a group of lysosomal storage disorders due to different lysosomal enzyme deficiencies, causing progressive storage of glycosaminoglycans (GAGs) in tissues and organs, and ultimately leading to multiorgan dysfunction [1]. GAG accumulation is progressive and, consequently, signs and symptoms of the disease worsen with age. Seven types of MPS are known due to 11 different enzyme deficiencies (Table 1). MPS II is the only one with X-linked inheritance, while all other MPS have an autosomal recessive transmission. Each MPS shows a wide spectrum of phenotypic variability, from the most severe form with signs/symptoms presenting at birth and severe cognitive delay to a milder form with signs/symptoms appearing in adulthood without intellectual impairment. This is mainly due to the different residual enzyme activity in each MPS patient which is related to the specific genetic dysfunction (for details, see the papers by Galimberti et al. [2], Rigoldi et al. [3] and Filocamo et al. [4] in this Supplement).

**Table 1:** The different types of mucopolysaccharidoses (MPS) with eponyms, enzymes, genes, loci, and glycosaminoglycans (GAGs) involved

MPS type	Subtype and eponyms	Deficient enzyme	Gene (locus)	GAGS involved
MPS I	Hurler (H)	α-l-iduronidase	IDUA (4p16.3)	Dermatan, heparan sulfate
	Hurler/Scheie (H/S)			
	Scheie (S)			
MPS II	Hunter A	Iduronate sulfatase	IDS (Xq28)	Dermatan, heparan sulfate
	Hunter B			
MPS III	Sanfilippo A	Heparan-N-sulfatase	SGSH (17q25.3)	Heparan sulfate
	Sanfilippo B	α-N-acetylglucosaminidase	NAGLU (17q21)	
	Sanfilippo C	Heparan acetyl-CoA:α-glucosaminide N-acetyltransferase	HGSNAT (8p11.1)	
	Sanfilippo D	N-acetylglucosamine 6-sulfatase	GNS (12q14)	
MPS IV	Morquio A	Galactose 6-sulfatase	GALNS (16q24.3)	Keratan, chondroitin sulfate
	Morquio B	β-galactosidase	GLB1 (3p21.33)	Keratan sulfate
MPS VI	Maroteaux-Lamy	Arylsulfatase B	ARSB (5q11-q13)	Dermatan sulfate
MPS VII	Sly	β-glucuronidase	GUS (7q21.11)	Dermatan, keratin, chondroitin sulfate
MPS IX		Hyaluronidase 1	HYAL (3p21.3)	Hyaluronan

It should be noted that the MPS V designation as Scheie syndrome is no longer used, Scheie syndrome now is the attenuated subtype of MPS I; the designation of MPS VIII was based on incorrect data

The most typical clinical phenotypes of MPS (Hurler, Hunter, and Morquio syndromes) were described about 100 years ago [5–7]. The identification of MPS III and MPS VI as separate clinical entities is more recent, occurring in the 1960s when it became possible to distinguish a single disease entity on the basis of the pattern of urinary GAGs [8, 9]. In those days, the pathophysiology of these disorders was far from being clarified and, at that time, it was debated whether they were caused either by increased synthesis or decreased degradation of GAGs [10]. Only gradually at the end of the 1960s did it become clear that MPS were due to a deficit of enzymes causing GAG storage. In 1964 van Hoof and Hers showed lysosomal enlargement in the hepatocytes of a Hurler patient [11] and in 1968 to 1969 Fratantoni et al. clearly demonstrated that Hurler and Hunter syndrome storage was corrected in vitro by incubating both fibroblasts in the same medium [12, 13]. This observation could only be explained by the lack of a degradative factor, probably a secreted diffusible enzyme, which was able to make a cross-correction of the defect. Scheie syndrome, although it was initially reported in 1962 as a “forme fruste of Hurler syndrome” [14], was later considered a separated entity [15]; in 1984 the demonstration of allelism of Hurler, Hurler-Scheie, and Scheie syndromes was made [16]. MPS VII, a very rare disease characterized by high frequency of hydrops fetalis and with a wide variability in severity, was described for the first time in 1973 by William Sly [17]; MPS IX, caused by hyaluronidase deficiency, was identified very recently in 1996 and only four patients have been described so far with features similar to juvenile rheumatoid arthritis [18].

Most of the enzyme deficiencies underlying these disorders were characterized between the 1970s and the 1980s [19] and identification of their coding genes came subsequently in the 1990s and first years of this century [19]. It then became possible to study the synthesis, modification, and transport of these enzymes in the various compartments of the cells and to search for effective therapies. The fact that lysosomal storage disorders were probably susceptible to replacement therapy was deducible from the demonstration of cross-correction made by Fratantoni et al. [12, 13], and was also envisaged by Prof. de Duve, the discoverer of lysosomes, in 1964 [20]: “It may be well to keep in mind that any substance which is taken up intracellularly in an endocytic process is likely to end up within lysosomes. This obviously opens up many possibilities for interaction, including replacement therapy”.

## Diagnosis

The suspicion of MPS might arise from clinical observation of typical signs and symptoms, but the confirmation of the diagnosis and the identification of the specific type of MPS are made in the laboratory where a biochemical evaluation of urinary GAGs and specific enzyme assays are performed and followed by molecular diagnosis (see Filocamo et al. in this Supplement [4]). Diagnosis of a metabolic disorder may also come from neonatal screening techniques. Among MPS disorders, MPS I is the most suitable to be included in neonatal screening programs because the advantage of an early diagnosis allowing early treatment is quite evident [21–24]. Pilot studies on MPS I screening are ongoing in many countries worldwide and in some regions of Italy (Toscana/Umbria and Veneto). Other treatable MPS (MPS II, IV, VI, and VII) will probably be included in screening pilot studies in the near future. Public health decisions to start neonatal screening for each disease should also take into consideration the ethical problems; neonatal screening involves apparently healthy babies and must face, for example, pseudodeficiency (an apparently reduced enzyme activity without clinical signs and symptoms) and genetic variants with uncertain meaning (see Donati et al. in this supplement [25]). The chance of false-positive results is not rare according to a recent paper [26]. As a consequence, physicians should learn how to modulate communication of the screening results to parents, and laboratories should give prompt results from second- and third-tier tests; psychological support services for the families are also needed. As with other groups of diseases, newborn screening for MPS raises the risk of overtreatment and, since all these are very rare diseases, evidence of its efficacy is not often statistically demonstrated but only “very likely” [27].

## Treatments for MPS

The presently available treatments for MPS have been effective in ameliorating the lives of individuals affected by these diseases, but are far from assuring a complete return to normalcy. In a recent interesting review, Grabowsky and Whitley outlined the ten (plus one) major challenges related to treatment of lysosomal storage disorders, most of them applying to MPS. They are, for example:

- the need to better understand the details of lysosomal functions within the cells;
- the mechanisms triggering secondary and tertiary events responsible for the propagation of disease;
- the tissue specificity-related mechanisms, including possible different tissue-specific therapeutic thresholds for efficacy;
- the biodistribution of therapeutic agents;
- the need for markers indicating when the “dysfunction” becomes irreversible tissue disruption; and
- the new disease manifestations consequent to a partial therapy, such as severe osteoarticular damage in MPS I-Hurler (MPS I-H) patients who underwent hematopoietic cell transplantation (HCT) [28].

The road to optimized medical care for MPS over the next years will certainly deal with these challenges.

Many papers in this Supplement are focused on treatments for MPS, including palliative (mainly surgeries), enzyme replacement therapy (ERT), and future treatments, including gene therapy. HCT was the first attempt at, and first successful treatment for, MPS dating back to the 1980s.

### Hematopoietic cell transplantation

The first etiologic treatment based on cross-correction was allogeneic HCT, first employed in a patient affected with MPS I-H in 1980 by Hobbs et al. [29]. Subsequently, more than 500 MPS I-H patients have been transplanted using this procedure [24]. All MPS together comprise around one-third of the transplanted patients affected by lysosomal storage diseases, with MPS I representing at least 70% of MPS [30]. Event-free survival in MPS I-H patients has much improved in the last 10–15 years thanks mainly to transplant protocol/guidelines which resulted in a significantly improved engraftment survival rate of over 90% in specialized centers [30]. At present, HCT is the first-line treatment for MPS I-H and is considered safe enough for recommendation, not only for Hurler patients but also severe Hurler/Scheie syndrome patients [31]. HCT is also the treatment of choice for patients who do not tolerate ERT or those with a logistic hindrance to the administration of regular infusions.

However, there is still a major concern regarding the significant residual disease burden observed over the long-term, primarily related to musculoskeletal features [32]. It is presently being discussed if the association of ERT with HCT for a long time after engraftment could help improve the musculoskeletal outcomes, and clinical studies are ongoing (www.clinicaltrials.gov identifier: NCT01173016, accessed 9 March 2018).

After the successful results obtained in MPS I-H, HCT was further performed in MPS II and III but, since it did not give the same satisfactory results on the cognitive outcome, this was abandoned [33–38]. Only recently has HCT been tested again in investigational studies in MPS II patients enrolled according to a more rigid protocol taking into account the age of enrollment [30]. From the more recent studies, it appears that HCT may also be successful in MPS II patients provided that it is performed quite early [39–41].

Few papers are available in the literature reporting results of HCT for MPS IV and MPS VI [42–48]. The reluctance to treat these patients with HCT is reasonably related to the high morbidity (graft failure, graft-versus-host disease, infection during immunosuppression, endocrine and gonadal failure) and mortality risks associated with HCT when faced with the lack of cognitive involvement (or very marginal involvement) in these two diseases. However, there are reports of good outcomes in patients who were intolerant or nonresponsive to ERT [45–47]. Combined treatment of HCT with ERT has also been reported with good results [48].

In summary, HCT is at present the only suitable treatment for brain disease; its morbidity and mortality risks have progressively reduced over the last 15 years, and indications have been consequently expanded. Investigational studies are ongoing on its possible application in MPS II. For MPS IVA and VI, evidence of efficacy is limited and, given the availability of ERT and the lack of prominent central nervous system (CNS) disease in these two MPS, the risk/benefit ratio of the procedure might still appear too high in these cases. Provided that the risks of HCT continue decreasing, its main advantages over ERT are a more physiological enzyme delivery to tissues, reduced cost, and one permanent treatment instead of continuous lifetime weekly hospital treatments.

### Enzyme replacement therapy

Fifteen years ago (in 2003) treatment with ERT became available for MPS I and was followed by MPS II and MPS VI in 2006, and MPS IV in 2014. ERT is indicated only for those MPS patients who do not have CNS involvement because, in the present formulation, these enzymes cannot pass through the blood–brain barrier. Details about each enzyme and the results of treatment are given in the paper by Concolino et al., in this Supplement [49].

### Future treatments

The papers by Fecarotta et al. [50] and Bellettato and Scarpa [51] in this Supplement, open up our minds to the many possible future directions of treatments based on our knowledge of pathophysiology. Delivery of drugs to the CNS, principles of substrate reduction, and the role of autophagy are the subjects of these papers, allowing us to appreciate the wide number of possible therapeutic approaches and their present development. Fraldi et al. ([52] in this Supplement) provide insights into the state-of-art accomplishments made with in-vivo and ex-vivo gene therapy-based approaches in MPS. Together, these three papers offer a clear vision of the future scenario of therapeutic challenges for MPS.

### Palliative treatments

The value of the multidisciplinary, complex, palliative treatments for MPS must never be overlooked. Their impact in improving health and survival of these patients is still relevant today. As mentioned earlier, present etiologic therapies may leave a significant residual burden which may partially be reduced with palliative therapies, with most of these consisting of surgical treatments. These treatments are not without risk, mainly in relation to the increased risks of anesthesiological treatments in these subjects (for details, see the paper by Moretto et al. in this supplement [53]), but also for the sometimes uncertain postsurgery results. The decisions about these treatments are time consuming and a source of doubt and discussion for the whole team treating the patient and for the family; they are also highly emotional, involving both the family and the medical team. However, surgery in some cases can change the course of the life of an individual with MPS allowing, for example, the patient to continue to walk instead of moving to a wheelchair. Everything is more complex in these patients than in healthy individuals and the success of surgical intervention depends mainly on the awareness, skill, and expertise of the treating team. Ear, nose, and throat (ENT), orthopedic, neurosurgical, ophthalmic, and cardiac treatments are all reviewed in this supplement (see papers by Bianchi et al. [54], Borgo et al. [55], Giussani et al. [56], Del Longo et al. [57], Boffi et al. [58]). The multidisciplinary team taking care of MPS patients consists not only of the surgeons, but also expert anesthesiologists, cardiologists, radiologists, and neuropsychologists (see papers by Moretto et al. [53], Boffi et al. [58], Spina et al. [59], Barone et al. [60], in this Supplement).

## Conclusions

This issue does not claim to cover all the clinical aspects related to MPS. As well as the lack of a review on HCT, there are other areas and arguments not covered, such as dental and maxillary issues [61, 62], treatment of tracheal narrowing and other deformities of the trachea [63–65], and the open question about the transition of care of the increasing number of pediatric patients to expert centers treating adult patients [66]. Nevertheless, we think it reaches the objective of helping those clinicians and specialists who have a diagnostic suspicion or are taking care of an MPS patient. Our aim was also to illustrate the complex task of taking care of an MPS patient, which implies the need for training, study, and multidisciplinary collaboration.

## Abbreviations

CNS: Central nervous system
ERT: Enzyme replacement therapy
GAG: Glycosaminoglycan
HCT: Hematopoietic cell transplantation
MPS I-H: Mucopolysaccharidosis I Hurler syndrome
MPS: Mucopolysaccharidosi(e)s
